# Altered Pain Perception in a Young Adult with Childhood Trauma and Suspected Riley-Day Syndrome: A Case Report

**DOI:** 10.3390/reports8020080

**Published:** 2025-05-26

**Authors:** Pedro Martínez-Lozano, Maurcio Sousa-Pitti, Natalia Toro-Pérez, Juan Nicolás Cuenca-Zaldívar, Rosana Cid-Verdejo, Oliver Martínez-Pozas, Laura Jiménez-Ortega, Eleuterio A. Sánchez-Romero

**Affiliations:** 1 Department of Physiotherapy, Faculty of Medicine, Health and Sports, Universidad Europea de Madrid, Calle Tajo, s/n, Urb. El Bosque, 28670 Madrid, Spain; pedro.martinez@universidadeuropea.es; 2 Interdisciplinary Research Group on Musculoskeletal Disorders, Calle Agustín Querol 5, 28014 Madrid, Spain; mausousapitti@gmail.com (M.S.-P.); natytp3516@gmail.com (N.T.-P.); rosana.cid@universidadeuropea.es (R.C.-V.); oliver.martp@gmail.com (O.M.-P.); 3 Grupo de Investigación en Fisioterapia y Dolor, Departamento de Fisioterapia, Facultad de Enfermería y Fisioterapia, Universidad de Alcalá, 28801 Alcalá de Henares, Spain; 4 Research Group in Nursing and Health Care, Puerta de Hierro Health Research Institute-Segovia de Arana (IDIPHISA), 28222 Majadahonda, Spain; 5 Physical Therapy Unit, Primary Health Care Center “El Abajón”, 28231 Las Rozas de Madrid, Spain; 6 Faculty of Dentistry, Universidad Complutense de Madrid, Plaza de Ramón y Cajal s/n, 28040 Madrid, Spain; 7 Department of Clinical Dentistry, Faculty of Biomedical Sciences, Universidad Europea de Madrid, Plaza de Francisco Morano s/n, 28670 Madrid, Spain; 8 Physiotherapy and Orofacial Pain Working Group, Sociedad Española de Disfunción Craneomandibular y Dolor Orofacial (SEDCYDO), 28009 Madrid, Spain; 9 Department of Psychobiology, Complutense University of Madrid (UCM), 28040 Madrid, Spain; laurajim@ucm.es; 10 Center for Human Evolution and Behavior, Instituto de Salud Carlos III (ISCIII), Complutense University of Madrid (UCM), 28029 Madrid, Spain; 11 Psychology and Orofacial Pain Working Group, Sociedad Española de Disfunción Craneomandibular y Dolor Orofacial (SEDCYDO), 28009 Madrid, Spain; 12 Department of Rehabilitation Sciences, Florida Gulf Coast University, Fort Myers, FL 33965, USA

**Keywords:** central sensitization, pain perception, Riley-Day syndrome, childhood trauma, psychopathology, case report

## Abstract

**Background and Clinical Significance**: Altered pain perception is a diagnostic challenge for patients with a history of trauma and substance use. Familial dysautonomia (Riley-Day syndrome) may further complicate the sensory profiles. **Case Presentation**: We describe a male in his late twenties, originally from Central America, with a history of severe childhood trauma and chronic cannabis use, who reported diminished pain perception despite multiple injuries. Despite the absence of nociceptive pain (nociceptive hypoesthesia), abnormal sensations, such as tingling and itching (paresthesia), and occasionally unpleasant burning sensations (dysesthesia) were common symptoms in this case. Diagnosis: Clinical suspicion of familial dysautonomia was raised based on altered pain perception and minor autonomic signs. However, no genetic testing or neurological evaluation was performed. Psychological assessment revealed high levels of neuroticism, depression, and maladaptive coping. The Central Sensitization Inventory (CSI) and the Symptom Severity Scale (SS) further supported the presence of psychological symptoms suggestive of possible central sensitization. Outcome: Functional improvement was observed after a reduction in substance use and implementation of self-directed physical and cognitive rehabilitation. No standardized follow-up or formal interventions were recorded. **Conclusions**: This case illustrates the complexity of pain modulation in trauma-affected individuals and emphasizes the need for an integrative, interdisciplinary evaluation of atypical pain presentations.

## 1. Introduction and Clinical Significance

Pain is a complex and subjective experience influenced by biological, psychological, and social factors [1]. Although most individuals exhibit a protective response to painful stimuli, a small number of cases present paradoxical reactions, including diminished or absent pain perception, despite significant tissue damage [2]. Such cases pose important diagnostic and therapeutic challenges, particularly when early psychological trauma, maladaptive coping, and possible genetic neuropathies converge to a single clinical profile [3].

This case report presents an unusual clinical picture of a young adult male with a history of childhood trauma and polysubstance use who exhibited subjective altered pain perception, high scores in central sensitization assessments, and significant emotional and behavioral dysregulation. Notably, a suspected diagnosis of Riley-Day syndrome (familial dysautonomia) is a rare hereditary sensory and autonomic neuropathy that may explain the patient’s diminished nociceptive sensitivity [4].

What makes this case particularly unique is the combination of altered pain perception and an apparent inability to feel pain, except for abnormal sensations such as paresthesia. This paradox challenges the conventional understanding of nociception and opens new avenues for research on the neurocognitive and emotional mechanisms underlying pain processing [5]. Furthermore, it underscores the need for integrative, trauma-informed, and interdisciplinary approaches to assessment and treatment planning in physiotherapy and mental health settings [6].

This document was written according to the CAse REport (CARE) guidelines for case reports [7]. The study was conducted in accordance with the guidelines of the Declaration of Helsinki, and written informed consent was obtained from all patients involved in the study. This study was reviewed and approved by the Research Ethics Committee for Medicines (CEIm) of the University Hospital of Getafe (approval number: CEIm25/61; 24 April 2025).

## 2. Case Presentation

### 2.1. Patient Information

#### 2.1.1. De-Identified Patient Data

The patient was a male in his late twenties, originally from Central America, and currently residing in Southern Europe. He was a daily smoker, a regular cannabis user, and a student of a health sciences program.

#### 2.1.2. Primary Concerns and Symptoms

The patient underwent psychological evaluation because of longstanding and atypical alterations in subjective pain perception and abnormal sensations, such as tingling and itching (paresthesia) and occasionally unpleasant burning sensations (dysesthesia), despite the absence of nociceptive pain (nociceptive hypoesthesia). In contrast, he experienced intermittent sharp pain in the ear and spontaneous pain episodes that disappeared when attention was directed toward them.

#### 2.1.3. Medical, Family, and Psychosocial History

The patient had a significant psychosocial history marked by severe childhood trauma, including physical and psychological abuse by his father, from a young age until early adolescence. He began using psychoactive and hallucinogenic substances at the age of 15 and engaged in high-risk behaviors during adolescence and early adulthood. Between the ages of 16 and 22, he regularly used synthetic LSD, mescaline, cannabis, MDMA, and other substances. He described this period as self-inflicted degradation linked to feelings of unworthiness and unresolved trauma.

Currently, he consumes approximately 2.5 g of cannabis daily despite experiencing periods of abstinence. He stated that drug use helps him to “think less” and feel emotionally detached. At 22 years of age, he was prescribed antipsychotics, antidepressants, and benzodiazepines. He is currently taking antidepressants, which are tapered off. Despite his psychiatric history, he described his current emotional state as being stable and fulfilling.

The patient reported no known family history of genetic disorders, although his mother was suspected of having symptoms consistent with Riley-Day syndrome. Several of his close friends died due to drug-related causes.

#### 2.1.4. Relevant Past Interventions and Outcomes

During the acute psychiatric phase, the patient received pharmacological interventions, but adherence was poor. He abruptly discontinued all medications and substances, leading to profound cognitive and functional decline, including loss of speech fluency, impaired reading comprehension, and difficulty with basic activities of daily living. A turning point occurred when he began physical training (calisthenics) and cognitive rehabilitation independently. Over time, he regained functional capacity and reported improved self-esteem and mood.

### 2.2. Clinical Findings

The patient underwent comprehensive clinical and psychological evaluations because of his reported insensitivity to pain and complex psychosocial history.

#### 2.2.1. Physical Examination and Medical History

Despite multiple documented physical traumas, including nasal septum fracture, vertebral disc injury, clavicle fracture, and a recent grade II ankle sprain, the patient reported little to no pain. Instead, he describes unusual sensory experiences such as pressure, localized tension, burning, and paresthesia. He exhibited hypersensitivity to cold stimuli and reported discomfort due to contact with an ultrasound probe. Cold hypersensitivity was evaluated by applying a cold spray of approximately 1 µm.

Repeated musculoskeletal ultrasound assessments of the upper and lower extremities, as well as the temporomandibular joints, revealed no current structural damage. The slump test reproduced symptoms indicative of increased neuronal mechanosensitivity, suggesting possible neuropathic pain, particularly in the lumbosacral region, resembling those previously associated with L2 vertebral injury and characterized by allodynia, tingling, and burning sensations. Four months after the ankle sprain, the patient still reported limited dorsiflexion, which did not interfere with daily functioning.

He also reported tinnitus in his left ear and perceived a reduction in physical performance, particularly during running, which he attributed to past injuries and possible long-term effects of substance use.

#### 2.2.2. Psychological Evaluation

##### Initial Interview

During the initial interview, the patient disclosed having had a difficult adolescence, including a history of childhood abuse, and his father physically assaulted him until the age of 13. He described his father as having an authoritarian and unstable personality, similar in some ways to his own, and indicated that they were frequently in conflict. The patient currently expresses remorse for behaviors between the ages of 16 and 22, when he regularly used synthetic LSD and mescaline, approximately twice daily, and engaged in high-risk activities such as selling marijuana. He rationalizes his drug use as a form of “relief/release” from depressive symptoms and suicidal ideation. He reported having obtained a firearm and considered suicide upon the anticipated death of his grandmother, who had cancer.

He smoked tobacco and cannabis (approximately 2.5 g per day). He was able to abstain from cannabis use for six months and has now resumed use for the past eight months, though he is contemplating cessation once again. He explains that drug use helps him avoid “overthinking” and being “too smart”.

At the age of 22, he was prescribed antipsychotics, antidepressants, and benzodiazepines. He is currently taking antidepressants only and is gradually tapering the dosage. He describes this period as one of the most stable and happiest times in his life. Despite not having family nearby, he found social support from a few close friends.

##### Psychological Questionnaire Results

Following the initial interview, several psychological assessments were administered to evaluate personality (NEO-FFI) [8], psychological symptomatology (SCL-90) [9], stress coping strategies (Coping Responses Inventory—Adult Form, CRI-A) [10], central sensitization inventory (CSI) [10], symptom severity (SS1 and SS2) [11], and cognitive performance (DOMINIO-40).

**Figure 1:**
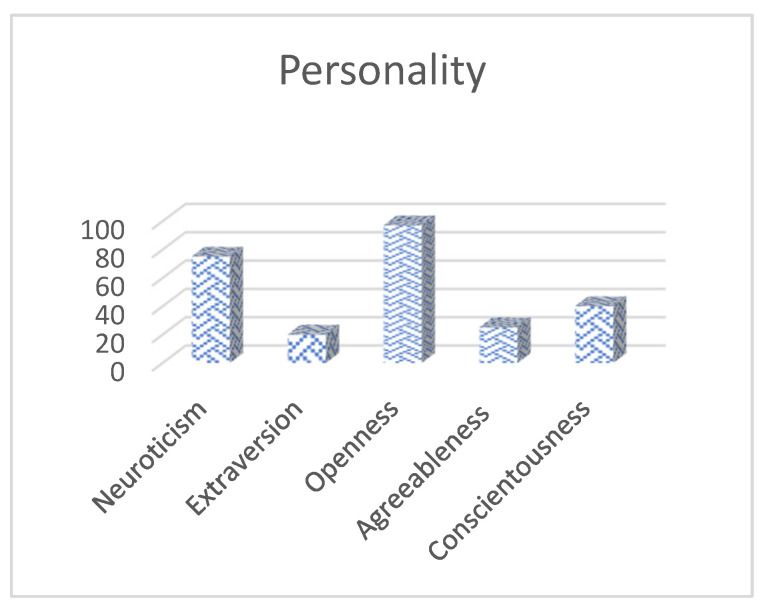
Percentile scores obtained by the patient in the five domains of the NEO-FFI personality questionnaire: neuroticism, extraversion, openness to experience, agreeableness, and conscientiousness. Percentiles are based on normative Spanish population data. Higher percentiles reflect the greater presence of each trait.

The NEO-FFI personality questionnaire revealed that MSP is present with a high level of neuroticism, very high openness to experience, low levels of extraversion and agreeableness, and an average level of conscientiousness (Figure 1). These results suggest that a person is prone to emotional instability and negative affect and is open to novel ideas, interests, and unconventional values. However, he appeared introverted, socially withdrawn, and somewhat individualistic and displayed limited trust in others. Additionally, he scored 29 on the intellectual ability, verbal comprehension, and abstract reasoning questionnaires, corresponding to the 50th percentile, which places him within the average range of the general population. However, it should be noted that frequent cannabis use may affect attention and memory, potentially affecting this result.

**Figure 2:**
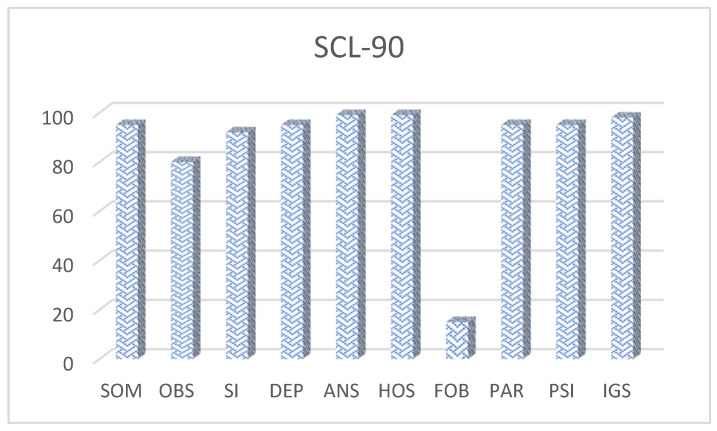
Percentile scores obtained by the patient in the nine dimensions of the SCL-90-R: somatization, obsessive–compulsive, interpersonal sensitivity, depression, anxiety, hostility, phobic anxiety, paranoid ideation, and psychoticism, plus the Global Severity Index (GSI). Scores above the 80th percentile indicate very high levels of psychological symptomatology.

In the SCL-90 questionnaire, which comprises nine dimensions of psychological symptoms, MSP shows percentile scores above the 80th percentile in all scales except for Phobic Anxiety (PHOB). This indicates high or very high levels of somatization (SOM), obsessive–compulsive symptoms (O-C), interpersonal sensitivity (IS), depression (DEP), anxiety (ANX), hostility (HOS), paranoid ideation (PAR), and psychoticism (PSY). Consequently, the Global Severity Index (GSI) is positioned at the 98th percentile (Figure 2), meaning that only 2% of the Spanish population would present with an equal or greater degree of psychological symptomatology, thus classifying it as a very high level.

**Figure 3:**
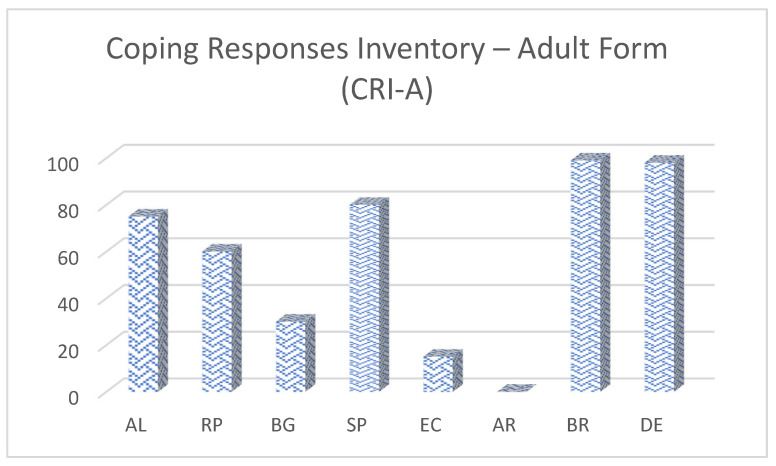
Coping profile of the patient based on the CRI-A inventory. Domains include logical analysis, positive reappraisal, guidance-seeking, problem-solving, cognitive avoidance, acceptance/resignation, emotional discharge, and alternative rewards. Higher values indicate greater reliance on that strategy when dealing with stress.

In the Coping Responses Inventory–Adult Form (CRI-A), the patient presented a very high level of alternative reward-seeking and emotional discharge, a high level of logical analysis and problem-solving, an average level of positive reappraisal, a very low level of acceptance and resignation, and low levels of guidance-seeking and cognitive avoidance (Figure 3).

Finally, both the Central Sensitization Inventory (CSI) and Symptom Severity (SS) questionnaires indicated the possible presence of central sensitization. The patient scored 50 on the CSI, and in the SS questionnaire, he reported experiencing more than 20 somatic symptoms, with a severity rating of 6.

##### Assessment and Conclusions

The patient presented with significant psychopathological symptoms, particularly depressive symptoms and a high degree of somatization. This elevated psychological distress, combined with a personality profile characterized by emotional instability and negative affectivity (neuroticism), shyness (introversion), a pronounced tendency to seek new experiences (openness to experience), and a coping strategy centered on the pursuit of alternative rewards when facing problems and family-related stressors, may have predisposed him to high levels of drug use and engagement in risky behaviors.

Moreover, regarding coping strategies in stressful situations (CRI-A), it is noteworthy that MSP exhibits an extremely low level of acceptance/resignation and a very high level of emotional discharge. While the latter may be adaptive in situations where problems cannot be solved, the overall profile suggests difficulty with passive acceptance and a tendency toward externalized emotional responses.

Currently, despite the questionnaire findings, the patient reports that he is in a stable period of life, studying physiotherapy and describing this as the happiest time he has experienced.

It is important to highlight that the patient reported an altered subjective pain perception (claiming not to feel pain but to experience paresthesia such as itching and burning). His standardized assessment scores indicated a high degree of central sensitization. Emotional disturbances, such as anxiety, depression, and somatization, also identified in this case, are commonly associated with impairments in nociceptive stimuli processing (i.e., nociceptive modulation) when evaluated using clinical tools such as quantitative sensory testing in populations with chronic pain [12,13,14]. In addition, previous research has identified a link between childhood adversity and impairments in pain processing [15], which is consistent with the patient’s severe paternal abuse during childhood. Therefore, it is reasonable to suggest that this patient may have experienced deficits in nociceptive processing, which could be reflected in the reduced pain perception as a result of changes in their psychological profile. Nonetheless, this study did not include a clinical assessment of nociceptive processing, nor did it investigate the potential correlation between the psychological profile and pain sensitivity. Therefore, it is plausible that patients may present with central sensitization, including emotional and cognitive alterations in areas involved in pain perception but with a marked inability to experience pain, except for paresthetic sensations. Additionally, previous research has identified a link between childhood adversity and the development of central sensitization [10], which is consistent with the patient’s severe history of paternal abuse during childhood. Taken together, this may represent a unique case of possible central sensitization but without the typical hyperalgesic response usually observed in such conditions.

#### 2.2.3. Pain and Sensory Assessment

The Central Sensitization Inventory (CSI) yielded a score of 50, which exceeded the clinical cut-off and suggested the presence of central sensitization. The Symptom Severity questionnaire (SS) revealed more than 20 somatic symptoms, with a severity rating of 6. These findings support the hypothesis of an altered pain processing system with the potential involvement of both peripheral and central pathways. Throughout the clinical evaluation, the patient consistently reported a lack of nociceptive awareness in response to tissue injury (nociceptive hypoesthesia), while describing abnormal sensations such as tingling, burning, and itching. These sensory phenomena should be differentiated: paresthesia refers to non-painful abnormal sensations (e.g., tingling or itching), whereas dysesthesia implies unpleasant or distressing sensations (e.g., burning) that may not be provoked by an external stimulus. This distinction is important for accurate interpretation of the patient’s altered pain profile. Nevertheless, CSI and SS are primarily linked to psychological comorbidities in individuals suffering from chronic pain conditions rather than accurately representing the actual functioning of pain processing systems [16,17]. Furthermore, their scores exhibited weak correlations or no associations with other clinical tools used to evaluate pain processing, such as quantitative sensory testing. Regarding the results obtained from the questionnaires, it is plausible that these findings were linked to the altered psychological profile exhibited by the patient rather than being indicative of central sensitization, which has yet to be substantiated in humans [18].

Although CSI and SS questionnaires are widely used screening tools in populations with chronic pain, they do not provide direct or objective evidence of central sensitization. The absence of quantitative sensory testing (QST), functional neuroimaging, or formal neurological examination in this case limits the ability to confirm the central sensitization hypothesis. Future clinical evaluations should incorporate these modalities to better characterize the sensory processing mechanisms involved.

### 2.3. Timeline

**Figure 4:**
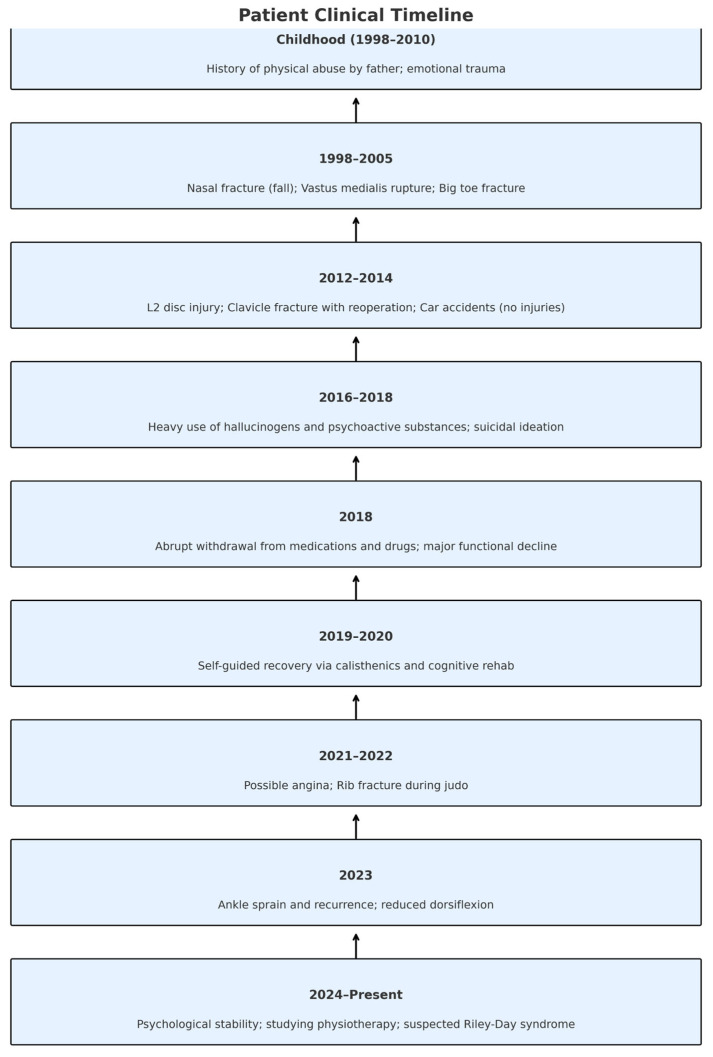
Chronological clinical timeline of the patient, summarizing major life events from childhood to present. Key milestones include episodes of trauma, onset of substance use, psychiatric decline, and gradual functional recovery through self-directed rehabilitation. This visual representation contextualizes the relationship between early adversity, altered pain perception, and the emergence of complex psychopathology, providing a temporal framework to interpret the current clinical profile.

The clinical, psychological, and behavioral history of the patient spans more than two decades and includes numerous physical injuries, psychiatric episodes, and periods of functional recovery. A visual representation of key events was created to facilitate understanding of the complex interplay between trauma, drug use, and altered pain processing (Figure 4).

### 2.4. Diagnostic Assessment

#### 2.4.1. Diagnostic Testing

The diagnostic process included a comprehensive psychological battery (NEO-FFI, SCL-90-R, CRI-A, CSI, SS questionnaires, and intelligence assessment) and a series of physical and functional evaluations, including musculoskeletal ultrasound imaging, neurodynamic testing (slump test), and sensory profiling. Imaging studies of previously injured areas, including the lower and upper extremities and temporomandibular joints (TMJ), revealed no current structural abnormalities.

The Central Sensitization Inventory (CSI) yielded a score of 50, exceeding the clinical threshold, suggesting possible central sensitization. The Symptom Severity Scale (SS) confirmed this suspicion, with the patient reporting more than 20 somatic symptoms and a severity score of 6. The SCL-90-R identifies severe psychopathological symptoms in multiple domains, particularly in depression and somatization. The NEO-FFI personality inventory revealed high neuroticism, low extraversion, agreeableness, and high openness to experiences. A cognitive test (DOMINIO-40) placed the patient in the 50th percentile for fluid intelligence.

#### 2.4.2. Diagnostic Challenges

Interpretation of the findings was complicated by the patient’s contradictory pain perception, marked by a near absence of nociceptive responses despite a history of significant physical injuries. This raises questions regarding possible congenital or acquired alterations in sensory pathways. Ongoing cannabis use may also have influenced cognitive testing results and pain reporting, although the patient demonstrated insight and active participation during the assessments.

Chronic cannabis use, particularly at high daily doses, has been associated with altered cognitive processing, emotional detachment, and disrupted interoceptive awareness, which may influence patients’ psychological test outcomes and pain self-reporting. While the patient subjectively associated cannabis use with emotional regulation and cognitive silencing, these effects may have confounded the interpretation of his altered pain perception and responsiveness.

#### 2.4.3. Diagnosis and Differential Considerations

While no formal genetic testing has been conducted to date, the clinical picture, marked by altered subjective pain perception, autonomic features (e.g., lack of sweating and cold hypersensitivity), and familial suspicion, led to the working hypothesis of familial dysautonomia (Riley-Day syndrome). This rare hereditary sensory and autonomic neuropathy (HSAN type III) might explain the dissociation between physical trauma and pain. Central sensitization, which is layered with high emotional reactivity and trauma-related dissociation, may further modulate the atypical sensory profiles.

#### 2.4.4. Prognosis

Currently, the prognosis is highly optimistic. The patient showed strong motivation, resumed academic study, and demonstrated functional stability. However, the persistence of central sensitization symptoms and emotional dysregulation warrants close multidisciplinary follow-up, including genetic evaluation and integrative care involving physical therapy and mental health support.

### 2.5. Therapeutic Intervention

#### 2.5.1. Type of Intervention

To date, no structured therapeutic interventions have been initiated in clinical rehabilitation programs. The patient engaged in a self-directed recovery process that combined physical activity, emotional self-regulation, and cognitive training. He reported that calisthenics-based physical training, which began after a period of functional decline, played a key role in his physical and psychological recovery. He also made a conscious effort to improve his reading comprehension and attention span as a part of his cognitive rehabilitation.

#### 2.5.2. Administration and Duration

These self-initiated interventions have been ongoing since 2019 with varying intensities. The patient maintained a physically active lifestyle and was enrolled in a physiotherapy degree program that served as a form of continuous functional and theoretical reinforcement. The patient continued to use antidepressants, which he was tapering under supervision. No physiotherapy or psychological therapy protocol was formally prescribed.

#### 2.5.3. Changes and Rationale

Over time, the patient reduced the use of psychoactive substances and eliminated all medications, except antidepressants. He also shifted from avoidance-based coping to more active and emotionally expressive strategies, as reflected in the CRI-A results. These changes appear to stem from his internal motivation to regain autonomy and functional capacity, and they coincide with the period he describes as “the most stable and happiest of his life”.

### 2.6. Follow-Up and Outcomes

#### 2.6.1. Clinician- and Patient-Assessed Outcomes

Although no standardized follow-up assessments were conducted by healthcare professionals, the patient reported significant improvements in emotional stability, motivation, and functional independence. He reported better management of emotional states, a more consistent daily routine, and renewed academic engagement. From the clinician’s perspective, the patient’s insight, verbal coherence, and capacity for reflection were notable during psychological evaluation.

#### 2.6.2. Follow-Up Tests and Findings

No additional imaging or laboratory tests were performed after the initial diagnosis. Ultrasound evaluation revealed no structural damage to the previously injured areas. Despite his history of repeated trauma, the absence of nociceptive responses remained unexplained, and a formal neurological evaluation, including genetic testing for suspected Riley-Day syndrome, was pending.

#### 2.6.3. Adherence and Tolerability

The patient demonstrated high adherence to self-directed therapeutic efforts, including regular physical activity and reduced substance use. The patient is currently receiving tapering antidepressant medications without any adverse effects. His adherence to academic responsibilities and social commitments further supported the stability of his current condition. To date, no indications of intolerance to any intervention have been reported.

#### 2.6.4. Adverse and Unanticipated Events

No adverse events occurred after the abrupt withdrawal of psychotropic medication in 2018, resulting in temporary cognitive and motor dysfunction. Since then, the patient has demonstrated steady improvement. The recent recurrence of ankle sprain during sports activity in 2023 was a minor setback, but it did not compromise his general recovery trajectory.

## 3. Discussion

### 3.1. Strengths and Limitations of the Case Report

This case offers a unique perspective on the intersection between psychological trauma, altered subjective pain processing, and suspected hereditary neuropathy. Patients’ self-awareness, functional recovery, and motivation for change represent strengths that support clinical optimism. Furthermore, the combination of possible central sensitization symptoms with an apparent absence of nociception challenges the conventional understanding of pain mechanisms and highlights the potential relevance of trauma-informed and neurodivergent approaches in rehabilitation.

Interestingly, paradoxical sensory profiles have been reported in neuropathic populations, where patients exhibit signs of sensitization, such as allodynia and hyperalgesia, despite not reporting pain. Forstenpointner et al. [15] found that sensory loss and hyperalgesia may coexist even in “painless” neuropathic patients, suggesting a dissociation between clinical symptoms and underlying sensory processing abnormalities. This underscores the potential relevance of central sensitization in patients with altered pain awareness.

However, this study has several notable limitations. First, the absence of formal genetic testing to detect biallelic pathogenic variants in ELP1 (formerly IKBKAP) [19] limits the ability to confirm a suspected Riley-Day syndrome diagnosis and the absence of other characteristic symptoms, such as postural hypotension and vomiting attacks, although poor motor coordination that provokes multiple traumatic damages may be related to it [20]. Second, the patient’s ongoing cannabis use may confound the interpretations of cognitive and sensory assessments. Furthermore, the lack of long-term clinical follow-up and standardized physical therapy interventions restricts the evaluation of structured outcomes over time. Finally, the absence of post-intervention clinician-assessed outcomes further limits the ability to determine the objective impact on the patient’s functional recovery.

Furthermore, no clinician-administered outcome measures or imaging studies were conducted following the patient’s self-directed rehabilitation efforts. Although he reported subjective improvements in emotional stability and functional capacity, the absence of objective follow-up assessments limited the strength of outcome inferences. Formal neurological referral and genetic evaluation are currently under consideration, and a multidisciplinary follow-up is recommended.

In light of the patient’s complex sensory presentation, we emphasize terminological precision when describing altered pain phenomena, distinguishing between nociceptive hypoesthesia, paresthesia, and dysesthesia.

### 3.2. Discussion of the Relevant Medical Literature

The patient’s clinical presentation aligns with theoretical frameworks describing the impact of early trauma and emotional dysregulation on pain perception and possible central sensitization. The literature has documented associations between childhood adversity and increased vulnerability to somatic syndromes, particularly in individuals with high neuroticism and maladaptive coping strategies.

Nanavaty et al. [21] conducted a systematic review that demonstrated a statistically significant link between psychological trauma, including childhood abuse, and increased pain sensitivity, with possible central sensitization identified as the key underlying mechanism.

Additionally, Delgado-Sanchez et al. [22] found that childhood trauma, particularly emotional abuse, was significantly associated with greater pain catastrophizing even after controlling for depression and anxiety. This supports the notion that personality traits such as neuroticism may amplify pain sensitivity and contribute to chronic pain syndromes.

Familial dysautonomia (Riley-Day syndrome) is a rare genetic disorder characterized by impaired pain and temperature sensations, autonomic instability, and emotional lability. Although most cases are diagnosed during childhood, atypical- or late-onset presentations have rarely been described. Milne et al. [23] reported the perioperative management of an adult patient with Riley-Day syndrome, reinforcing that although uncommon, adult presentations require careful multidisciplinary planning.

Moreover, the paradoxical coexistence of impaired pain processing and asymmetry or pain unawareness raises questions regarding the cortical processing of nociceptive inputs and dissociative mechanisms in traumatized individuals. Gerrans [24] proposed that such dissociative pain experiences can be interpreted through a hierarchical model of self-representation and allostatic regulation, offering a neuroscientific explanation for conditions such as pain asymmetry in trauma-exposed populations. The effect of regular cannabis use on pain perception and psychological functioning should be considered when interpreting patient responses. Cannabis-related modulation of attentional focus and affective processing could have contributed to the dissociation from pain and the paradoxical sensory profile observed.

In support of this, Mickleborough et al. [25] used functional magnetic resonance imaging (fMRI) to demonstrate that trauma-related cues in patients with post-traumatic stress disorder (PTSD) significantly altered pain processing in the brain, correlating with increased activation in regions linked to the dissociation and emotional modulation of pain.

### 3.3. Scientific Rationale and Interpretation

This may reflect a disruption in the integration of somatosensory inputs and emotional-affective processing. A patient’s preserved capacity to describe pain-related sensations (e.g., tingling and burning) without reporting pain may be linked to altered interoception or cortical modulation of pain signals. These phenomena are often under-recognized in physiotherapy but may critically inform intervention planning, particularly in patients with a history of trauma.

Scarpina et al. [26] demonstrated that interoceptive sensibility, specifically low trust and heightened emotional awareness, was significantly associated with increased pain sensitivity in women with vulvodynia, highlighting the relevance of interoceptive processing and cortical modulation in pain perception.

Psychological evaluation suggested that the patient presented with high emotional instability (neuroticism) and increased levels of psychological symptoms (SCL-90). However, regardless of the increased levels of emotional discharge and low acceptance, the patient also presented active behavioral coping strategies at the time of evaluation, implying a positive aptitude for recovery.

The interplay between early trauma, emotional dysregulation, maladaptive coping strategies compounded by chronic cannabis use, and potential neurodevelopmental anomalies may produce a neurophysiological context in which pain perception is fragmented or paradoxical. In this patient, such interactions may underlie the observed disconnection between physical injury and nociceptive awareness, supporting the multifactorial hypothesis that bridges affective neuroscience, sensory processing, and autonomic function.

Nonetheless, these interpretations should be considered exploratory and hypothesis-generating. As a single case study, this report could not establish causality or confirm the specific pathophysiological mechanisms. Further investigation with objective measures (e.g., QST, functional neuroimaging, and genetic testing) is essential to validate the proposed associations.

### 3.4. Main Takeaway Lessons

This case highlights the need to assess pain using a multidimensional lens, which includes emotional trauma, personality structure, coping strategies, and potential genetic factors. This underscores the value of combining psychological assessment with somatic evaluation in physiotherapy and mental health practices. Clinicians should be aware of paradoxical pain presentations, particularly in patients with a history of trauma or complex symptom profiles.

Although this case provides insights into the complex interactions between psychological, neurobiological, and behavioral factors in altered pain perception, the observations remain specific to a single individual. Therefore, caution is warranted when extrapolating these findings, and they should serve as a stimulus for further research rather than a basis for clinical generalization.

**LEARNING POINTS**
  - Altered pain sensitivity may coexist with diminished pain awareness in patients with psychological trauma.
  - Cannabis use can modulate both cognitive performance and interoceptive awareness, impacting clinical assessments.
  - Familial dysautonomia remains a clinical hypothesis when hallmark signs or genetic confirmation are lacking.
  - Trauma-informed and neurodivergent approaches may be essential in understanding paradoxical pain profiles.
  - Multidisciplinary follow-up is recommended to validate diagnostic hypotheses in complex chronic pain cases.

## 4. Patient Perspective

*“For a long time, I thought I deserved to suffer. Drugs were my escape and also my punishment. I wasn’t looking for fun—I wanted to destroy myself. I didn’t believe I deserved help or even kindness. I used to feel disconnected from my body, as if pain was not real, or not mine.”*

*“When I hit bottom, I lost many abilities—speaking, reading, even walking properly. But something changed in me. I decided to fight to get everything back. Training helped me feel alive again. I started to read, move, and think clearly again. It took time, but now I feel stable, maybe for the first time in my life.”*

*“Studying physiotherapy is part of my healing. I want to understand the body, pain, and how movement connects us to ourselves. I know my case is unusual, but maybe that’s why it can help others. I’m not fully recovered, and I still have questions about my body and my mind. But now, at least, I want to keep asking those questions.”*

## Data Availability

Data supporting the findings and conclusions are available upon request from the corresponding authors.
